# Using advanced cell models for targeted radionuclide therapy evaluation: increased efficacy in 3D versus 2D

**DOI:** 10.1186/s13550-026-01393-0

**Published:** 2026-04-03

**Authors:** Maria J. Klomp, Sigrun E. Erkens, Lilian van den Brink, Wytske M. van Weerden, Simone U. Dalm

**Affiliations:** 1 Department of Radiology & Nuclear Medicine, Erasmus MC, Rotterdam, The Netherlands; 2 Department of Urology, Erasmus MC, Rotterdam, The Netherlands

**Keywords:** Targeted radionuclide therapy, Three-dimensional, Spheroids, [^177^Lu]Lu-PSMA-I&T, Prostate cancer

## Abstract

**Background:**

Preclinically, radiopharmaceuticals are currently mainly evaluated in two-dimensional (2D) cell models, which lack clinical features that are relevant for accurate evaluation of targeted radionuclide therapy (TRT) responses. The development of three-dimensional (3D) cell models in the last years offers opportunities to overcome at least parts of the limitations of 2D cell models, but such 3D cell models are currently only rarely used in nuclear medicine research. Moreover, the comparison between 2D and 3D cell models, aimed at demonstrating the potential added value of 3D cell models, is even more scarce. To fill this gap and promote the use of more clinically relevant 3D cell models in nuclear medicine research, we performed such a comparative study. For this, we developed and evaluated 3D cell models derived from the prostate-specific membrane antigen (PSMA)-expressing human cancer cell lines LNCaP and PC3-PIP, by culturing these cells in anti-adhesive round bottom plates (“bio-spheroids”) or by culturing LNCaP cells in Matrigel (MG) or Noviogel-P5K (NG) domes (”MG-spheroids” or “NG-spheroids”). Hereafter, PSMA expression levels and [^111^In]In-PSMA-I&T uptake were determined and compared between 3D and 2D cell models. Additionally, we assessed cell viability of 3D- versus 2D-cultured cells after external beam radiation therapy (EBRT) and PSMA-TRT using [^177^Lu]Lu-PSMA-I&T.

**Results:**

No significant differences in viability were observed between bio-spheroids versus 2D cell models after EBRT and PSMA-TRT, neither in LNCaP nor in PC3-PIP cells. In contrast, LNCaP MG-spheroids had a significantly better response to PSMA-TRT in comparison to the 2D-cultured cells. This was despite a lower PSMA expression level and lower [^111^In]In-PSMA-I&T uptake in the MG-spheroids. Importantly, no significant difference in radiosensitivity was observed between these MG-spheroids and the 2D cell model.

**Conclusions:**

Despite lower PSMA expression levels and lower radiopharmaceutical uptake in LNCaP MG-spheroids, and albeit similar radiosensitivity, PSMA-TRT induced a stronger reduction in viability in the MG-spheroids in comparison to the 2D-cultured cells. In contrast, no differences were observed in PSMA-TRT efficacy between bio-spheroids and the 2D cell model. The aforementioned leads to the hypothesis that biological factors important for TRT, e.g. cross-radiation and radiopharmaceutical retention within the 3D cell structure, are better represented in MG-spheroids where cell-cell interactions are already formed prior to radiopharmaceutical incubation.

**Supplementary Information:**

The online version contains supplementary material available at 10.1186/s13550-026-01393-0.

## Background

Targeted radionuclide therapy (TRT), using radiopharmaceuticals directed against biomarkers overexpressed on cancer cells for the precise delivery of cytotoxic radiation, is clinically approved for the treatment of neuroendocrine tumors (NETs) and prostate cancer (PCa) [1, 2]. The beta-emitting radiopharmaceuticals [^177^Lu]Lu-[DOTA-Tyr^3^]octreotate and [^177^Lu]Lu-PSMA-617, which bind to somatostatin type-2 receptors (SSTR2) overexpressed on NETs and prostate-specific membrane antigen (PSMA) overexpressed on PCa, respectively, are used. The success of the abovementioned radiopharmaceuticals triggered further development of both novel and improved radiopharmaceuticals for known and new targets, and the evaluation of various not yet (commonly) applied radionuclides, such as actinium-225 which emits alpha-particles [3] and terbium-161 which emits beta-minus particles, conversion electrons and Auger electrons [4]. Additionally, (pre)clinical studies are focusing on improving TRT efficacy by developing combination treatment strategies with, amongst others, chemotherapy [5], DNA repair inhibitors [6], and immune checkpoint inhibitors [7].

Two-dimensional (2D) cell culture models are often used to evaluate radiopharmaceuticals in vitro. However, it is widely acknowledged that these 2D models do not accurately reflect the physical condition of tissues, e.g. cell-cell interactions are limited, cell-extracellular matrix (ECM) interactions are lacking and cell morphologies are altered. These limitations of 2D cultures can affect processes such as cell differentiation and proliferation, gene and protein expression profiles, and cell secretion and signaling [8, 9]; all which can also potentially affect TRT responses. Moreover, unlike what is often observed in the clinical situation, 2D-cultured cells have easy access to a rich environment of nutrients and oxygen, which results in the absence of necrosis induced by starvation and hypoxia. More unique to TRT, 2D cultures fail to reflect radiopharmaceutical diffusion and potentially underestimate the impact of cross-radiation, the latter being a process in which radioactivity bound to target-expressing cells also irradiates surrounding cells that do not express the target. The extent of cross-radiation depends on the penetration depth of the applied radionuclide and the process is especially of interest for tumors with heterogeneous target expression [10].

The development of more advanced three-dimensional (3D) cell culture models has opened the opportunity to overcome the aforementioned limitations associated with 2D cell culture models [11–13]. However, despite the growing technical possibilities, 3D cell culture models are still poorly used for investigating the efficacy of TRT [14–20], and the comparison to results obtained with 2D-cultured cells is often beyond the research scope. To move towards a more accurate evaluation of TRT efficacy, we aimed to compare TRT efficacy in 3D versus 2D cell culture systems. For this, we focused on PSMA-TRT and obtained 3D cell structures derived from PSMA-expressing PCa cell lines using two different methods. For accurate data interpretation of the observed PSMA-TRT responses in 3D and 2D, we analyzed PSMA expression levels, radiopharmaceutical uptake and radiosensitivity to EBRT.

## Methods

### Cell culture

Endogenously prostate-specific membrane antigen (PSMA)-expressing LNCaP cells (American Type Culture Collection) and PSMA-transduced PC3-PIP cells (kindly provided by prof. Anna Orlova, Uppsala University, Sweden) were used. These human PCa cell lines were cultured in RPMI-1640 + L-Glutamine (Gibco) supplemented with 5% (*v/v*) fetal bovine serum (FBS; Gibco), 100 U/mL penicillin and 100 µg/mL streptomycin (Lonza), and RPMI-1640 + GlutaMAX-I (Gibco) supplemented with 10% (*v/v*) FBS, 100 U/mL penicillin and 100 µg/mL streptomycin (Sigma-Aldrich), respectively. PC3-PIP cells were cultured with an additional 2 µg/mL puromycin every other week. Both cell lines were routinely passaged and cultured at 37 °C in a humidified atmosphere with 5% CO_2_.

### Radiolabeling

According to previously described methods [21], PSMA-I&T acetate (piCHEM) was radiolabeled with [^111^In]InCl_3_ (^111^In) (Curium Netherlands BV) or non-carrier added lutetium-177 (^177^Lu) (PI Medical) at a molar activity of 20 MBq/nmol or 60 MBq/nmol, respectively, with radiochemical yields > 95%. Moreover, [^177^Lu]Lu-DTPA was prepared as previously described [22] and used as a specificity control to demonstrate PSMA-specificity of results obtained using [^177^Lu]Lu-PSMA-I&T.

### Cell viability after PSMA-TRT and EBRT: bio-spheroids versus 2D

**Fig. 1:**
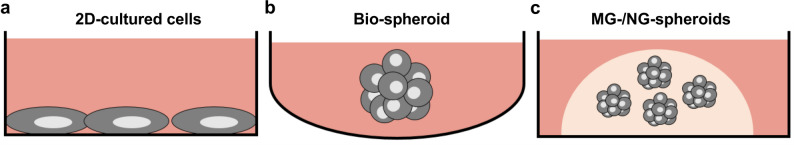
Schematic overview of the used cell culture systems, i.e. (**a**) 2D-cultured cells, (**b**) a bio-spheroid and (**c**) MG/NG-spheroids. *MG = Matrigel*,* NG = Noviogel*,* 2D = two-dimensional*

To determine the viability after PSMA-TRT of LNCaP and PC3-PIP cells, 500.000 single cells were resuspended in 1.0 mL incubation medium (i.e. RPMI-1640 + GlutaMAX-I supplemented with 1% (*w/v*) bovine serum albumin (Sigma-Aldrich), 20 mM HEPES (Sigma-Aldrich), 10% (*v/v*) FBS, and 0.06 mg/mL kolliphor HS 15 (a surfactant applied to prevent sticking)) containing [^177^Lu]Lu-PSMA-I&T or [^177^Lu]Lu-DTPA. Six activity concentrations of [^177^Lu]Lu-PSMA-I&T or [^177^Lu]Lu-DTPA were tested, i.e. between 0-2.0 MBq/mL for LNCaP and 0–0.5 MBq/mL for PC3-PIP. Lower activity concentrations were selected for PC3-PIP cells as these cells have higher PSMA expression levels resulting in higher radiation doses being delivered to the cells. LNCaP cells and PC3-PIP cells were incubated for 4–22 h, respectively, on a 3D rocking shaker at 37 °C, after which the cells were washed twice with cell culture medium via centrifugation (5 min, 200 x g, 4 °C). The incubation time was shortened to 4 h for LNCaP cells as 22 h incubation led to the formation of aggregates, hampering accurate execution of the following steps. Following centrifugation, the resulting cell pellets were resuspended in normal growth medium and plated both in (1) high-grade anti-adhesive BIOFLOAT 96-well plates (Sarstedt) (i.e. 7.000 LNCaP cells/well/100 µL and 250 PC3-PIP cells/well/100 µL) forming a single spheroid per well (further referred to as “bio-spheroids”), and in (2) clear-bottom white 96-well plates (Greiner) for 2D cell growth (i.e. 3.500 LNCaP cells/well/100 µL and 250 PC3-PIP cells/well/100 µL) (Fig. 1a and b). Six days after cell plating, pictures were taken to measure bio-spheroid surface area and the bio-spheroids were transferred to a clear-bottom white 96-well plate. Cell viability was determined by performing a CellTiter-Glo^Ⓡ^ 3D (Promega) assay according to manufacturer’s instructions for both 3D- and 2D-cultured cells.

Additionally, to determine the radiosensitivity of LNCaP and PC3-PIP cells, 2D-cultured cells at 80% confluence were exposed to different doses of EBRT (0–4.0 Gy) using the RS320 (Xstrahl Live Sciences; 1.6554 Gy/min, 195 kV, 10 mA). After collection by trypsinization, the cells were re-plated and analyzed in 3D and 2D as described above.

### MG/NG-spheroids formation

In addition to using LNCaP and PC3-PIP bio-spheroids, LNCaP spheroids were generated in 24-well plates by culturing 25.000 cells in a 40 µL dome of Matrigel^®^ (MG) (Corning) or 50.000 cells in a 40 µL dome of the polyisocyanopeptide hydrogel Noviogel-P5K (NG) (5 mg/mL, diluted 2.8 times in medium prior to use, Noviocell) (Fig. 1c), further referred to as “MG-spheroids” or “NG-spheroids”, respectively. MG is derived from mouse sarcoma cells and contains extracellular matrix components, i.e. mainly laminin and collagen IV, and tumor-derived proteins including growth factors, with batch-to-batch variability in protein content [23]. NG, on the other hand, is a synthetic, tunable and thermo-sensitive polyisocyanopeptide hydrogel, mimicking collagen [24]. As MG-spheroids formed more efficiently compared to NG-spheroids, probably due to the biological origin of MG, a lower cell number was plated. After seven days, the generated MG/NG-spheroids were photographed and used in follow-up experiments.

### Measuring spheroid surface area

The surface area of bio-spheroids and MG/NG-spheroids was determined using ImageJ software. The bio-spheroid surface area after PSMA-TRT and EBRT was obtained by measuring three technical replicates from two and three independent biological experiments, respectively. The surface area of LNCaP MG/NG-spheroids was derived from two independent biological replicates, analyzing thirty LNCaP spheroids from two different locations (fifteen per location) per biological replicate.

### Radiopharmaceutical uptake studies

To determine the uptake of [^111^In]In-PSMA-I&T in 2D-cultured LNCaP cells, cells were plated on poly-L-lysine (Sigma-Aldrich) coated 24-well plates (140.000 cells/well for 1 h and 4 h incubation; 70.000 cells/well for 22 h incubation). The next day, cells were washed twice with phosphate-buffered saline (PBS) and subsequently incubated for 1 h, 4–22 h with 0.5 mL incubation medium supplemented with 1 nM [^111^In]In-PSMA-I&T (0.02 MBq/mL), with or without 1 µM unlabeled PSMA-I&T. After incubation, cells were washed with cold PBS, trypsinized and collected. Subsequently, the number of cells was measured using the Countess II automatic cell counter (Invitrogen) and radiopharmaceutical uptake was determined using a gamma counter (HIDEX). To determine [^111^In]In-PSMA-I&T uptake in LNCaP MG/NG-spheroids, hydrogel-embedded spheroids were incubated with the radiopharmaceutical similarly as 2D-cultured cells and subsequently washed twice with PBS for 10 min. Afterwards, 0.5 mL TrypLE^TM^ Express Enzyme (Gibco) with 10 µM Rho-associated protein kinase (ROCK) inhibitor (AdipoGen Life Sciences) was added, the hydrogel dome structures were broken down by resuspension and single cells were collected after incubating the spheroids for 25 min at 37 °C. Cells were then washed twice with 0.5 mL incubation medium via centrifugation (5 min, 200 x g, 4 °C). The resulting cell pellet was resuspended in normal cell culture media, and the number of cells and radiopharmaceutical uptake was measured as described above. Uptake of [^111^In]In-PSMA-I&T is expressed as percentage added dose per 100.000 cells (% AD/100.000 cells).

### RT-qPCR

To measure *PSMA* mRNA expression levels, total RNA was isolated from LNCaP cells cultured in 2D and as MG-spheroids using the TRIzol extraction method. Subsequently, 500 ng RNA was converted into cDNA using the RevertAid First Strand cDNA Synthesis Kit (Thermo Scientific) according to manufacturer’s instructions. Afterwards, an RT-qPCR was performed by mixing 1 µL cDNA and 4 µL 1x SensiFAST SYBR^®^ Lo-ROX supplemented with 0.5 µM of both the forward and reverse primer. The QuantStudio 7 Flex RT-qPCR system with the QuantStudio Real-Time PCR software v1.5 was used and the thresholds to determine the Ct values were set at 0.15 for all genes. For analysis, the number of copies was determined by 2^(40−Ct)^, followed by dividing the number of PSMA copies over the geometric mean of the number of copies of three reference genes (i.e. β-Actin, GAPDH, HPRT1). An overview of the used primers can be found in Supplemental Table 1.

### Western blot analysis

To measure PSMA protein expression levels, LNCaP cells cultured in 2D and as MG-spheroids were lysed using RIPA buffer supplemented with protease inhibitor, and 10 µg of the cell lysates were run on 6% sodium dodecyl sulfate-polyacrylamide gels and subsequently blotted on polyvinylidene fluoride membranes. After blocking with 5% (*w/v*) non-fat dried milk for 1 h, the membrane was incubated overnight at 4 °C with the primary antibody, followed by incubation with the secondary antibody for 1 h at room temperature. The signal was visualized using the BM chemiluminescence western blot substrate (Roche), and PSMA monomers and β-actin were imaged (Amersham AI600). An overview of the used antibodies can be found in Supplemental Table 2.

### Cell viability after PSMA-TRT and EBRT: MG-spheroids versus 2D

Cell viability of LNCaP MG-spheroids was determined by incubating dome-embedded spheroids with 1.0 mL incubation medium with varying activity concentrations (0–2.0 MBq/mL) of [^177^Lu]Lu-PSMA-I&T or [^177^Lu]Lu-DTPA. After 22 h of incubation, samples were washed twice with PBS and 0.5 mL TrypLE™ Express Enzyme with 10 µM ROCK inhibitor was added. Unlike what was done in the radiopharmaceutical uptake studies where single cells were collected after incubation at 37 °C, MG-spheroids were collected without incubation at 37 °C to preserve their 3D structure. The spheroids were then washed with incubation medium via centrifugation (5 min, 750 x g, 20 °C), re-suspended in cell culture medium and 10.000 cells/well/100 µL were re-plated on MG-coated clear-bottom white 96-well plates to ensure the 3D cell structure is maintained. To calculate the required volume of cell suspension, single cells from separate domes were obtained and counted. The viability of LNCaP cells in 2D cultures following PSMA-TRT was assessed by plating cells one day before start of the assay in poly-L-lysine-coated 96-well plates, after which 170 µL incubation medium with identical activity concentrations of [^177^Lu]Lu-PSMA-I&T or [^177^Lu]Lu-DTPA (0–2.0 MBq/mL) was added. After 22 h of incubation, cells were washed with cell culture medium. For both 3D- and 2D- cultured cells, a CellTiter-Glo^Ⓡ^ 3D viability assay was performed after 6 days.

Moreover, the radiosensitivity of LNCaP MG-spheroids and 2D-cultured LNCaP cells towards EBRT was compared. Regarding radiosensitivity in 3D, MG-spheroids were exposed to different EBRT doses (0–4.0 Gy), followed by re-plating the spheroids as described above and a CellTiter-Glo^Ⓡ^ 3D assay 6 days after irradiation. The obtained viability was compared to that of 2D-cultured LNCaP cells following EBRT, as previously outlined. Examples of irradiated LNCaP MG-spheroids sub-cultured on MG-coated well plates are provided in Supplemental Fig. 1, demonstrating that, amongst others, the 3D structure is well-maintained after re-plating.

### Statistics

The Graphpad 9.0.0 software was used for statistical analysis. Both technical and biological outliers were removed. Subsequently, the normality of the data was tested with a Shapiro-Wilk test, followed by either a parametric or non-parametric unpaired t-test or a one-way ANOVA with a Tukey’s multiple comparison post-hoc test, to test for differences in spheroid surface area, radiopharmaceutical uptake and PSMA expression level. Moreover, for each activity concentration/dose applied during viability studies, a two-way ANOVA with a Šidák correction was performed to test for differences between 3D- and 2D-cultured cells. Results are considered significant when the *p*-value is less than 0.05. All results represent the mean ± standard deviation (SD). The detailed results of the analyses, i.e. mean, SD and *p*-values, can be found in Supplemental Tables 3–9.

## Results

### Cell viability after PSMA-TRT and EBRT: bio-spheroids versus 2D

**Fig. 2:**
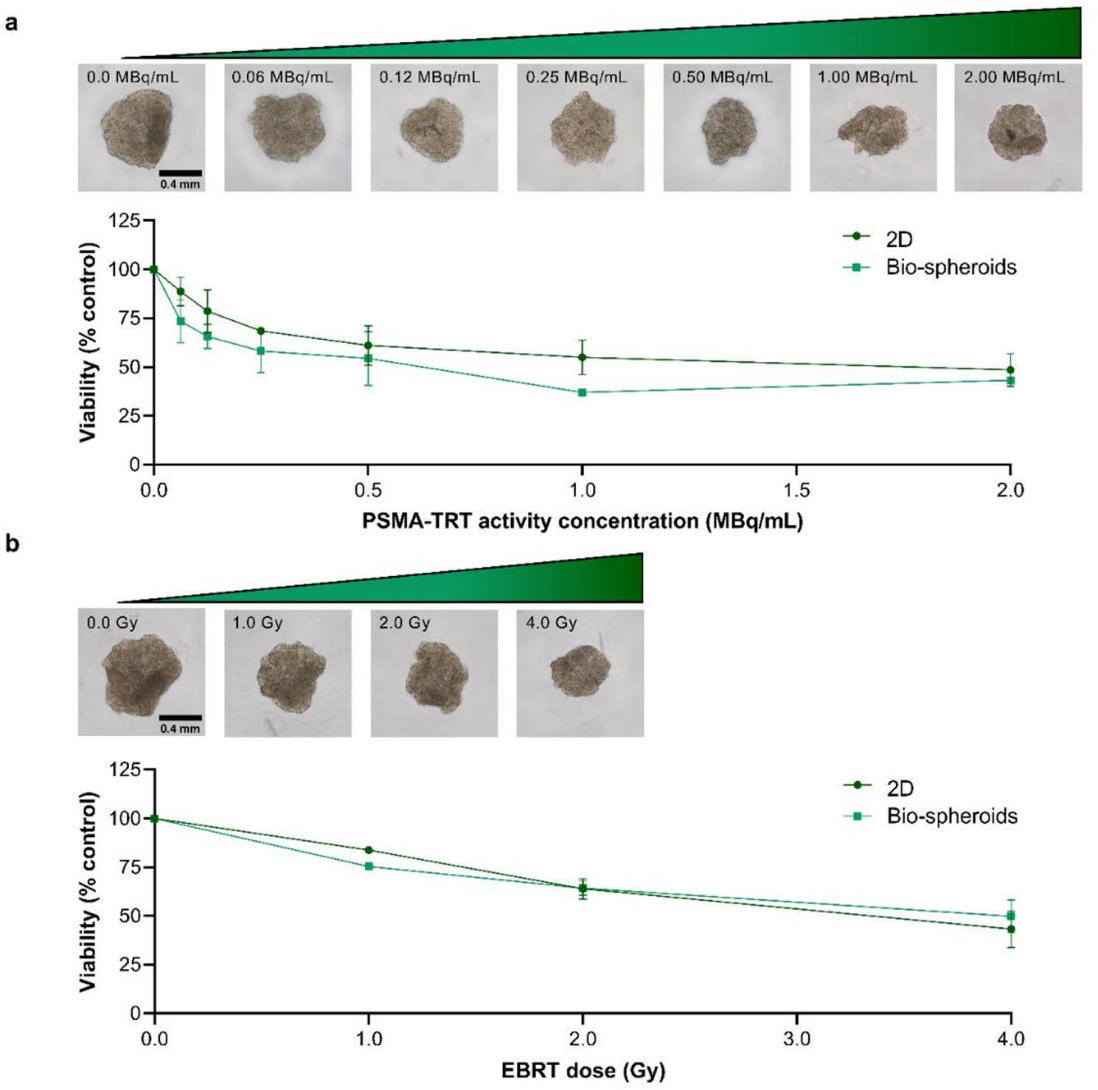
Viability of LNCaP cells cultured as bio-spheroids or in 2D setting measured 6 days after (a) incubation with several activity concentrations of PSMA-TRT (n = 3) or (b) exposure to varying doses of EBRT (n = 3). Data are normalized to untreated controls. Representative images of untreated, PSMA-TRT-treated and EBRT-treated bio-spheroids are shown. All microscopic images have the same scale. *2D = two-dimensional*,* PSMA-TRT = prostate-specific membrane antigen targeted radionuclide therapy*,* EBRT = external beam radiation therapy*,* Gy = gray*

After incubating a suspension of single cells with [^177^Lu]Lu-PSMA-I&T in a suspension of single cells, LNCaP cells were plated either in anti-adhesive well plates to obtain bio-spheroids or in 2D setting. Regarding bio-spheroids, a dose-dependent reduction in spheroid surface area and viability was observed after radiopharmaceutical incubation (Fig. 2a, Supplemental Fig. 2a). A similar reduction in cell viability was observed in 2D-cultured LNCaP cells (Fig. 2a), e.g. 54.4 ± 13.8% versus 61.0 ± 10.2% viable cells in 3D- and 2D-cultured cells, respectively, after treatment with 0.5 MBq/mL [^177^Lu]Lu-PSMA-I&T (*p* = 0.95). Similar activity concentrations of non-targeting [^177^Lu]Lu-DTPA resulted in a significantly higher viability for (at least) the two highest tested activity concentrations (i.e. 1.0–2.0 MBq/mL) in both bio-spheroids and 2D-cultured cells (Supplemental Fig. 3a and b), demonstrating that the observed reduction in cell viability was the result of PSMA-mediated radiopharmaceutical binding.

Consistent with the observed PSMA-TRT responses, surface area and cell viability of LNCaP bio-spheroids were reduced dose-dependently after EBRT (Fig. 2b, Supplemental Fig. 2b). Reduction in cell viability of 2D-cultured LNCaP cells after EBRT was in the same range (Fig. 2b), e.g. 64.2 ± 3.6% viable cells in 3D setting and 63.9 ± 5.2% viable cells in 2D after treatment with a dose of 2.0 Gy (*p* > 0.99), indicating an absence of differences in radiosensitivity.

**Fig. 3:**
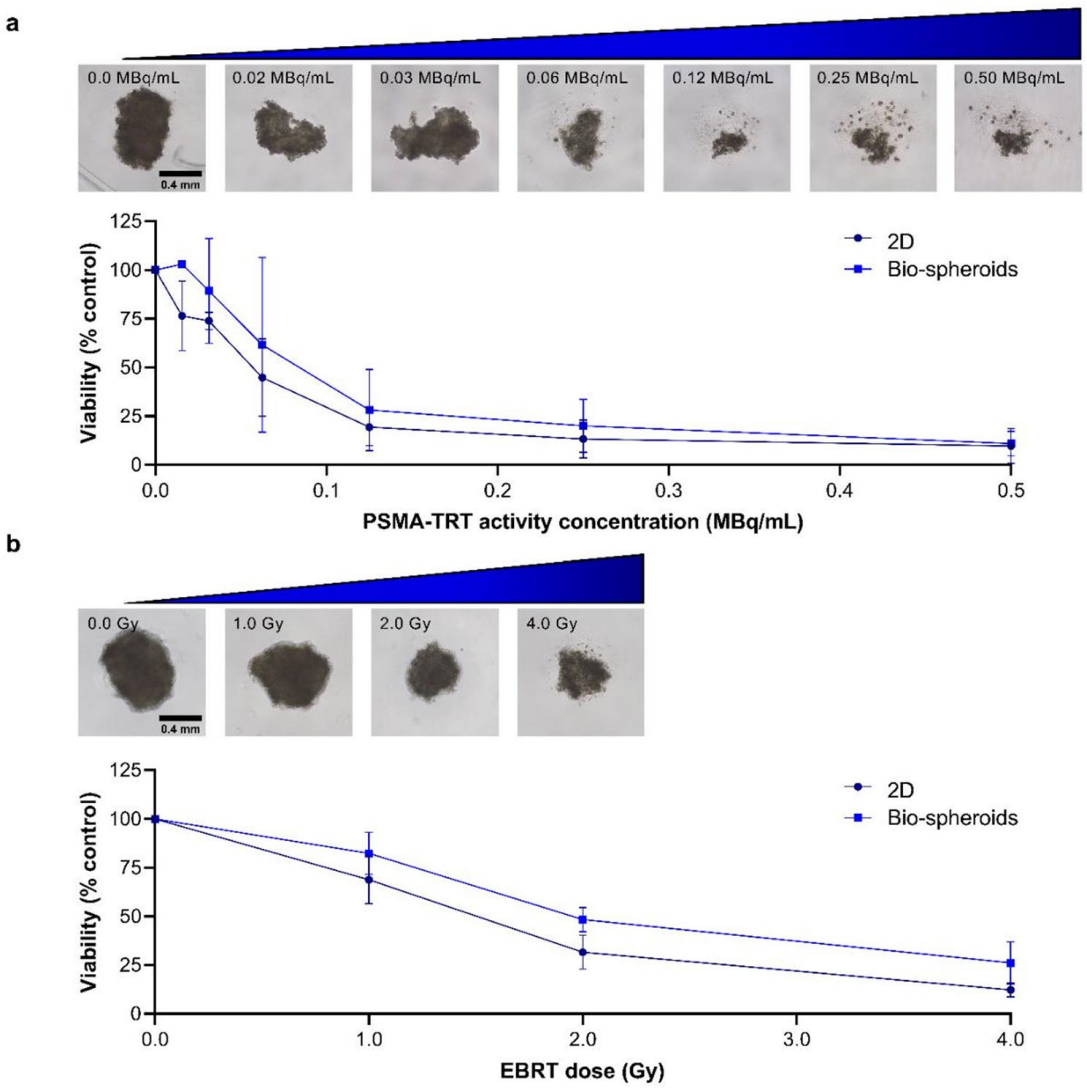
Viability of PC3-PIP cells cultured as bio-spheroids or in 2D setting measured 6 days after (a) incubation with several activity concentrations of PSMA-TRT (n = 4) or (b) exposure to varying doses of EBRT (n = 3). Data are normalized to untreated controls. Representative images of untreated, PSMA-TRT-treated and EBRT-treated bio-spheroids are shown. All microscopic images have the same scale. *2D = two-dimensional*,* PSMA-TRT = prostate-specific membrane antigen targeted radionuclide therapy*,* EBRT = external beam radiation therapy*,* Gy = gray*

Similar experiments were performed with PC3-PIP cells, demonstrating dose-dependent reductions in bio-spheroid surface area and viability after PSMA-TRT (Fig. 3a, Supplemental Fig. 2c), without significant differences in cell viability between bio-spheroids and 2D-cultured cells, e.g. 89.2 ± 26.9% versus 73.9 ± 4.5% viable cells in 3D and 2D setting, respectively, after 0.03 MBq/mL [^177^Lu]Lu-PSMA-I&T (*p* = 0.92) (Fig. 3a). Treatment with non-targeting [^177^Lu]Lu-DTPA resulted in higher cell viabilities for the highest activity concentrations (Supplemental Fig. 3c and d), demonstrating that the observed reduction in cell viability after PSMA-TRT was PSMA-dependent.

The SD of the viability data of TRT-treated LNCaP cells cultured in 2D or as bio-spheroids (Fig. 2a) is visually smaller than that observed for PC3-PIP cells (Fig. 3a), especially at lower activity concentrations. Therefore, the independent biological experiments of TRT-treated PC3-PIP cells, both cultured in 2D and 3D setting, are separately visualized in Supplemental Fig. 4. This demonstrates that the variance does not arise from differences between 2D- versus 3D-cultured cells, but rather from variability between biological replicates, potentially caused by the high proliferation rate of PC3-PIP cells.

In line with the above, EBRT reduced PC3-PIP cell viability to a similar extent in bio-spheroids and 2D-cultured cells (Fig. 3b), e.g. 48.4 ± 6.3% and 31.6 ± 8.6% viable cells in 3D and 2D, respectively, after treatment with 2.0 Gy EBRT (*p* = 0.08). Similarly, a dose-dependent reduction in bio-spheroid surface area was observed (Fig. 3b, Supplemental Fig. 2d).

### Radiopharmaceutical uptake and PSMA expression: MG/NG-spheroids versus 2D

**Fig. 4:**
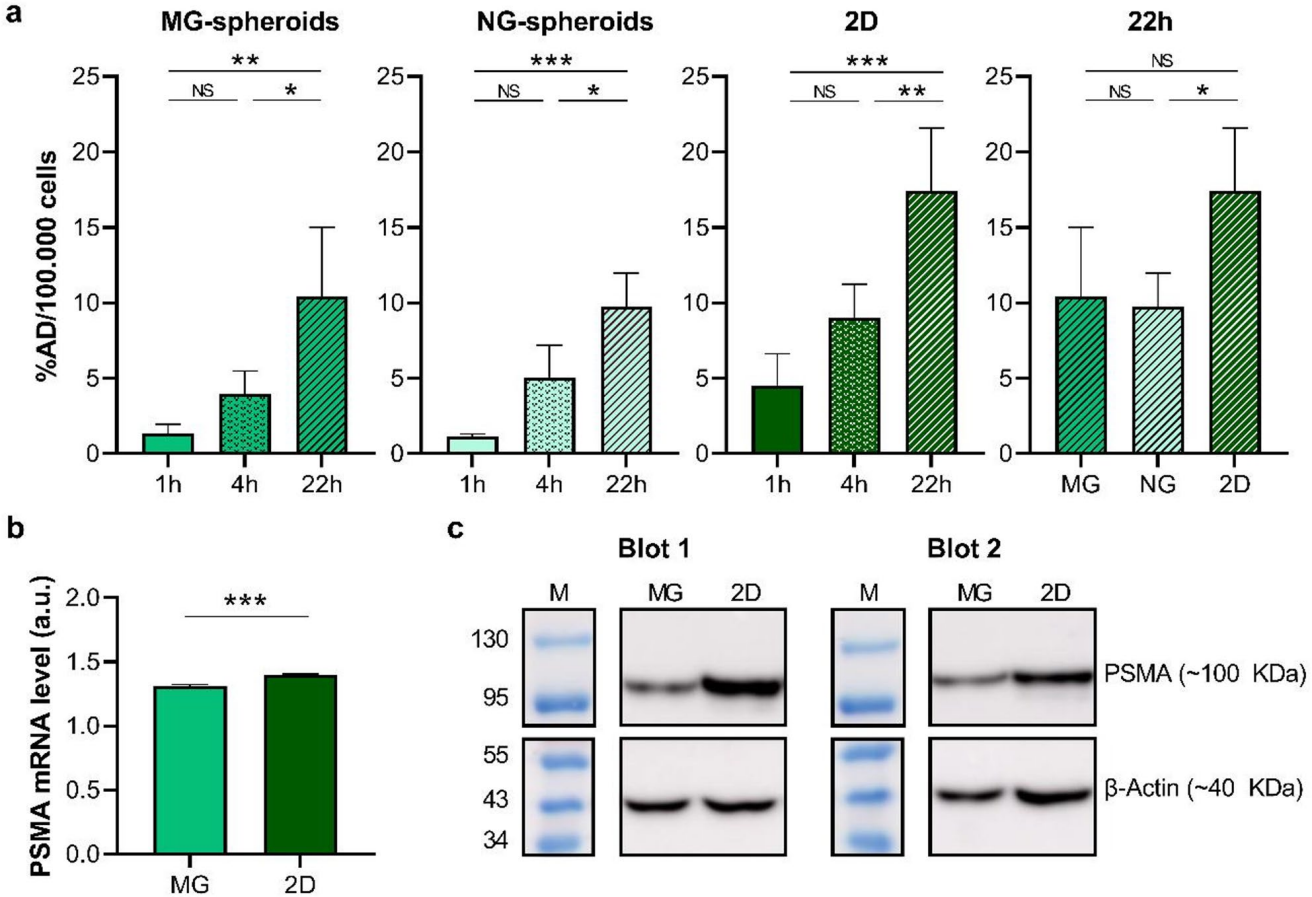
(**a**) Uptake of [^111^In]In-PSMA-I&T by LNCaP cells cultured as MG/NG-spheroids (*n* = 4) or in 2D setting (*n* = 5) after 1 h, 4 h and 22 h of radiopharmaceutical incubation. (**b**) *PSMA* mRNA (*n* = 3) and (**c**) PSMA protein (*n* = 2) expression levels of LNCaP cells cultured as MG-spheroids (*MG*) or as monolayer (*2D*). Asterisks indicate (**a**) significant differences in radiopharmaceutical uptake over time or between different culture systems, tested using a one-way ANOVA with a Tukey’s multiple comparison post-hoc test, or (**b**) significant differences in *PSMA* mRNA expression between MG-spheroids and 2D-cultured cells, as tested by a parametric unpaired t-test. *% AD / 100.000 cells = percentage added dose per 100.000 cells*,* 2D = two-dimensional*,* MG = Matrigel*,* NG = Noviogel*,* a.u. = arbitrary units*,* h = hours*,* M = protein marker. NS = non-significant*, * p  < 0.05, ** p  < 0.01, *** p < 0.001

Besides bio-spheroids, LNCaP cells were cultured as MG/NG-spheroids in hydrogel domes (Supplemental Fig. 5a). The radiopharmaceutical uptake was equal for MG- and NG-spheroids, i.e. [^111^In]In-PSMA-I&T uptake in MG-spheroids was 1.4 ± 0.6%, 4.0 ± 1.5% and 10.4 ± 4.6% AD/100.000 cells at 1 h, 4 h and 22 h of incubation, respectively, versus 1.1 ± 0.2%, 5.1 ± 2.1% and 9.8 ± 2.2% AD/100.000 cells in NG-spheroids, respectively (Fig. 4a), despite significant differences in the average surface area for the MG- versus NG-spheroids (Supplemental Fig. 5b). For comparison, the [^111^In]In-PSMA-I&T uptake in 2D-cultured LNCaP cells was 4.5 ± 2.1%, 9.0 ± 2.2% and 17.4 ± 4.2% AD/100.000 cells after 1 h, 4 h and 22 h of incubation, respectively (Fig. 4a). For both the MG/NG-spheroids and 2D-cultured cells, the uptake increased significantly upon extension of the incubation period from 4 h to 22 h (*p* = 0.02 for MG/NG-spheroids, *p* = 0.002 for 2D). At 22 h of incubation, no difference in uptake was found between the spheroids cultured in the two different hydrogels (*p* = 0.97), whereas the MG- and NG-spheroids had a 40.2% (*p* > 0.05) and 43.9% (*p* = 0.03) lower uptake, respectively, in comparison to 2D-cultured cells. Since no difference in radiopharmaceutical uptake was observed between MG- and NG-spheroids, and because MG-spheroids are formed more efficiently (Supplemental Fig. 5b), likely due to the natural origin of this hydrogel, we selected MG for follow-up experiments.

Subsequently, PSMA expression of LNCaP MG-spheroids and 2D-cultured cells was assessed. In line with differences in radiopharmaceutical uptake, it was demonstrated that *PSMA* mRNA expression levels were significantly reduced by 6.6% in MG-spheroids compared to 2D-cultured LNCaP cells (*p* < 0.001, Fig. 4b). Related hereto, PSMA protein expression levels were lower for MG-spheroids as well (Fig. 4c, Supplemental Fig. 6).

### Cell viability after PSMA-TRT and EBRT: MG/NG-spheroids versus 2D

**Fig. 5:**
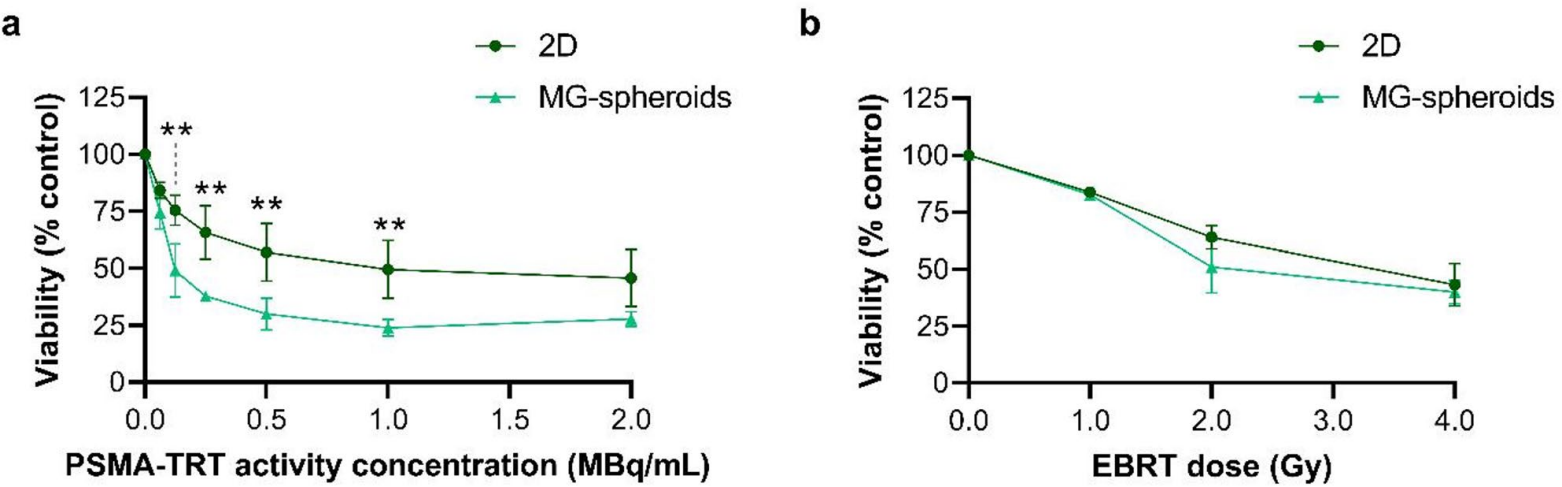
Viability of LNCaP cells cultured as MG-spheroids or in 2D setting measured 6 days after treatment with (**a**) several activity concentrations of PSMA-TRT (*n* = 3 for MG, *n* = 4 for 2D) or (**b**) varying doses of EBRT (*n* = 3). Data are normalized to untreated controls. Asterisks indicate significant differences in cell viability after an identical activity concentration of PSMA-TRT between MG-spheroids versus 2D-cultured cells, tested using a two-way ANOVA with Šidák correction. *2D = two-dimensional*,* MG = Matrigel*,* PSMA-TRT = prostate-specific membrane antigen targeted radionuclide therapy*,* EBRT = external beam radiation therapy*, Gy = gray. ** p  < 0.01

Subsequently, the response to PSMA-TRT and EBRT was assessed in LNCaP MG-spheroids and compared to 2D-cultured LNCaP cells. Six days after [^177^Lu]Lu-PSMA-I&T incubation, a dose-dependent reduction in cell viability was observed in MG-spheroids with statistical significant differences compared to 2D for several activity concentrations (Fig. 5a), e.g. 30.0 ± 6.9% and 57.1 ± 12.6% viable cells in 3D and 2D setting, respectively, after treatment with 0.5 MBq/mL [^177^Lu]Lu-PSMA-I&T (*p* = 0.002). Importantly, non-targeting [^177^Lu]Lu-DTPA had only a limited effect on cell viability at all tested activity concentrations (Supplemental Fig. 7a and b).

In contrast to cell viability after TRT, a similar radiosensitivity in LNCaP MG-spheroids versus 2D-cultured cells was observed (Fig. 5b), e.g. 51.1 ± 11.4% and 63.9 ± 5.2% viable cells, respectively, after treatment with an EBRT dose of 2.0 Gy (*p* = 0.06).

## Discussion

Radiopharmaceuticals are preclinically mainly evaluated using 2D in vitro cell culture models. This is despite the fact that 2D models have several limitations that are important for accurate evaluation of TRT efficacy. Currently, the benefits of 3D cell culture models to better capture the biology and physiology of tissues have gained interest for assessing TRT efficacy. In this study, we investigated the response of PCa cell line-derived 3D culture models to PSMA-TRT and compared the response to that observed in 2D cultures.

Only few studies have been published using 3D cell culture models to assess radiopharmaceuticals. Spheroids derived from NET cell lines (i.e. wild-type and/or SSTR2-transfected BON-1 and QGP-1 cells, and wild-type NCI-H727 cells) were generated for evaluating the response to SSTR2-TRT using [^177^Lu]Lu-DOTATATE or [^67^Cu]Cu-EB-TATE [14–16]. In addition to a (dose-dependent) effect on spheroid size, Purohit et al. [14] and Njotu et al. [15] reported an increase in DNA damage markers, proliferation markers and/or cell death (markers). For PSMA-TRT of PCa, studies reported a dose- and/or spheroid radius-dependent reduction in growth [17–20] and/or viability [20] using a wide variety of PCa cell lines, e.g. C4-2(B), LNCaP(-LN3), PC3(-PIP) and DU 145. In the current study, we demonstrated an enhanced PSMA-TRT effect in LNCaP MG-spheroids compared to the effect in 2D-cultured cells. However, unlike this, we did not observe a difference in PSMA-TRT efficacy of bio-spheroids versus 2D-cultured cells. This indicates that, using the methods we have applied, hydrogel-embedded spheroids have benefit over bio-spheroids, and might take factors relevant for TRT evaluation better into account.

Hydrogel-embedded spheroids were obtained by culturing LNCaP cells in either the biological-derived MG or the synthetic hydrogel NG. Despite differences in the source of the hydrogel and the surface area of LNCaP MG- versus NG-spheroids, no significant difference was found in radiopharmaceutical uptake. A higher uptake was observed in 2D-cultured LNCaP cells, which could, at least partly, be explained by the higher *PSMA* mRNA and PSMA protein expression levels measured in the 2D cultures versus the MG-spheroids. Additionally, a potential limited diffusion of the radiopharmaceutical through the hydrogel needs to be taken into account for MG/NG-spheroids as also reported for hormones and chemotherapeutics [25]. It is important to investigate this aspect in follow-up studies, e.g. by performing high-resolution in vitro autoradiography studies evaluating radiopharmaceutical uptake throughout the complete spheroid after multiple incubation time points.

We observed an enhanced PSMA-TRT efficacy in LNCaP MG-spheroids compared to 2D-cultured cells, despite the lower radiopharmaceutical uptake for the 3D-cultured cells. Additionally, the measured viability after EBRT showed no difference in radiosensitivity between the two systems. This is in contrast to findings obtained with LNCaP cells described in literature [26]. Of note, both the method to obtain 3D cell structures and the readout measurement are different in the aforementioned study, which could be the reason for this discrepancy. Taken together, the lower radiopharmaceutical uptake and the similar response to EBRT indicates that additional factors, specific for the method used for obtaining MG-spheroids where strong cell-cell interactions are formed prior to radiopharmaceutical incubation, contribute to a more effective PSMA-TRT response in this system. Such factors could include enhanced cross-radiation and improved retention of the radiopharmaceutical within the MG-spheroid.

Hydrogel-embedded spheroids were only generated using LNCaP cells. This experimental approach was also tested with PC3-PIP cells, but was unfortunately not successful, i.e. spheroids partly disintegrated during re-plating, resulting in 2D cell growth. Future studies assessing TRT efficacy in PC3-PIP MG-spheroids might have to be developed with spheroids remaining in MG after radiopharmaceutical incubation. Of note, it will then be important to evaluate whether the gel itself does not retain the radiopharmaceutical.

In contrast to the difference in PSMA-TRT efficacy between LNCaP MG-spheroids and 2D-cultured cells, there was a lack of difference between bio-spheroids and 2D-cultured cells. PSMA expression levels and PSMA-targeted radiopharmaceutical uptake levels were similar in both culture systems, as cells were plated in 2D and 3D using the same cell suspension after radiopharmaceutical incubation. It was proven that culturing the cells as bio-spheroids or in 2D did not affect the cancer cells’ radiosensitivity to EBRT. We hypothesize that the factors contributing to higher PSMA-TRT responses in MG-spheroids are not contributing to the improved treatment efficacy in bio-spheroids. Since the bio-spheroids are formed after radiopharmaceutical incubation, unlike in the hydrogel-embedded setting, cell-cell interactions still need to be formed, which most likely limits cross-radiation and radiopharmaceutical retention within the 3D cell structure.

It would be of value to further optimize the experimental set-up and, instead of incubating single cells is suspension followed by re-plating, incubate the bio-spheroids with the radiopharmaceutical. This requires that experiments be initiated when the bio-spheroids are relatively small in size, enabling accurate assessment of spheroid viability several days after TRT or EBRT. Due to the small spheroid size at the start of the assay, we experienced spheroid disintegration and/or loss of the spheroid, preventing further analyses.

Raitanen et al. [20] also compared PSMA-TRT efficacy in LNCaP spheroids with the response observed in 2D setting. They demonstrated that, despite a lower radiosensitivity of 3D-cultured cells in comparison to 2D-cultured cells as described in one of their other studies [26], 3D-cultured cells were more sensitive to PSMA-TRT compared to 2D cultures. Based on Monte Carlo simulations, the authors hypothesized that this was caused by geometric effects leading to a higher dose for 3D-cultured cells compared to 2D-cultured cells. Of note, radiopharmaceutical uptake and retention were not taken into account during these simulations. In our study, we demonstrated that the increased response to TRT in MG-spheroids is not due to higher radiopharmaceutical uptake or increased radiosensitivity of the cells. This led to the hypothesis that factors such as cross-radiation and radiopharmaceutical retention are more accurately modelled in these 3D structures compared to conventional 2D cell cultures. This can mainly be of interest when comparing TRT efficacy with a radiopharmaceutical coupled to radionuclides with different linear energy transfers. The lack of cell-cell interactions in all directions in conventional 2D systems is likely to introduce biases for such studies. Moreover, MG-spheroid cultures may provide insights into the cellular cross-talk between cells that are affected by PSMA-TRT, changing the microenvironment and inducing a bystander effect. Such studies can be extended to characterizing the relevance of a hypoxic gradient resulting in a necrotic core [27] and heterogeneity of PSMA expression within the spheroid [28]. Moreover, increasing the complexity of MG-spheroids by co-culturing cancer cells with other cell types, such as stromal cells and immune cells, is of high added value. Together, this would contribute to a more clinically representative model, allowing evaluation of PSMA-TRT effects on the tumor as a whole, rather than solely direct cell damage of targeted cells. In addition to studying PSMA-TRT, such models would also allow the study of radiopharmaceuticals targeting stromal targets, e.g. radiopharmaceuticals targeting fibroblast activation protein-α present on cancer-associated fibroblasts [29]. However, despite these novel research opportunities, 3D cell culture models are not required to address relatively simple research questions. For example, 2D-cultured cells are highly valuable and sufficient for assessing affinity and specificity of radiopharmaceuticals, as well as studying agonistic versus antagonistic properties.

## Conclusions

In conclusion, our study shows the feasibility of evaluating PSMA-TRT efficacy in 3D cell culture models, and demonstrates that in 3D models, especially hydrogel-embedded spheroids where cell-cell interactions are established prior to radiopharmaceutical uptake, there is a significant difference in PSMA-TRT response compared to 2D cell cultures. Overall, this highlights the new era of using more biologically relevant models for radiopharmaceutical evaluation, especially for studies focused on TRT efficacy. Future studies should investigate whether this difference in TRT efficacy is also observed in other PCa models, as well as in NET models.

## Supplementary Information

Below is the link to the electronic supplementary material.

Supplementary Material 1

## Data Availability

The generated data is available upon reasonable request by reaching out to the corresponding author.

## Abbreviations

2D: Two-dimensional
3D: Three-dimensional
EBRT: External beam radiation therapy
ECM: Extracellular matrix
FBS: Fetal bovine serum
MG: Matrigel
NETs: Neuroendocrine tumors
NG: Noviogel
PBS: Phosphate-buffered saline
PCa: Prostate cancer
PSMA: Prostate-specific membrane antigen
PSMA-TRT: Prostate-specific membrane antigen targeted radionuclide therapy
ROCK: Rho-associated protein kinase
SD: Standard deviation
SSTR2: Somatostatin type-2 receptor
TRT: Targeted radionuclide therapy
