# Supplementary material for: Using advanced cell models for targeted radionuclide therapy evaluation: increased efficacy in 3D versus 2D
Source: EJNMMI Res. 2026 Apr 3;16:56. doi: 10.1186/s13550-026-01393-0 (PMC13062042; doi:10.1186/s13550-026-01393-0)
Supplement: Supplementary file 1 — Supplementary Material 1 [file 13550_2026_1393_MOESM1_ESM.pdf]

Supplementary information (SI)

Title

Using Advanced Cell Models for Targeted Radionuclide Therapy Evaluation: Increased Efficacy in 3D versus 2D

Author names

Maria J. Klomp[1], Sigrun E. Erkens[2], Lilian van den Brink[1], Wytske M. van Weerden[2], Simone U. Dalm[1].

Affiliations

1. Department of Radiology & Nuclear Medicine, Erasmus MC, Rotterdam, The Netherlands
2. Department of Urology, Erasmus MC, Rotterdam, The Netherlands.

Corresponding author

Dr. Simone U. Dalm, s.dalm@erasmusmc.nl

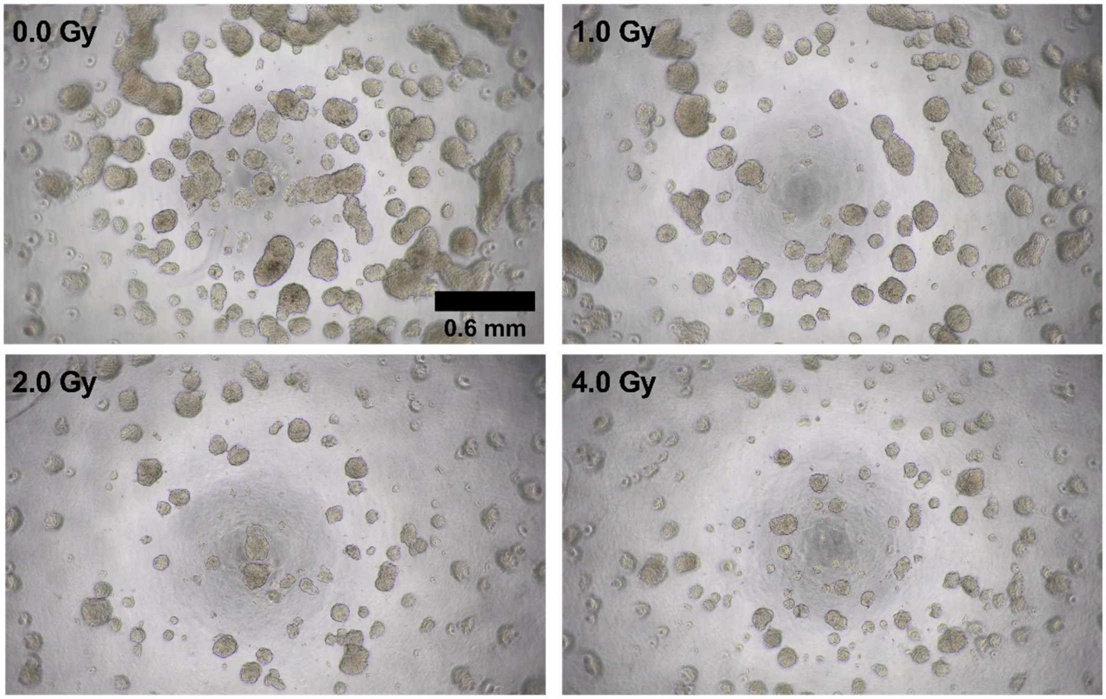

Supplemental Fig. 1 Representative images of LNCaP cells cultured as MG-spheroids and re-plated on MG-coated well plates after treatment with varying doses of EBRT, demonstrating that, amongst others, the 3D structure is well-maintained after re-plating. All microscopic images have the same scale. *3D = three-dimensional, EBRT = external beam radiation therapy, Gy = gray, MG = Matrigel*

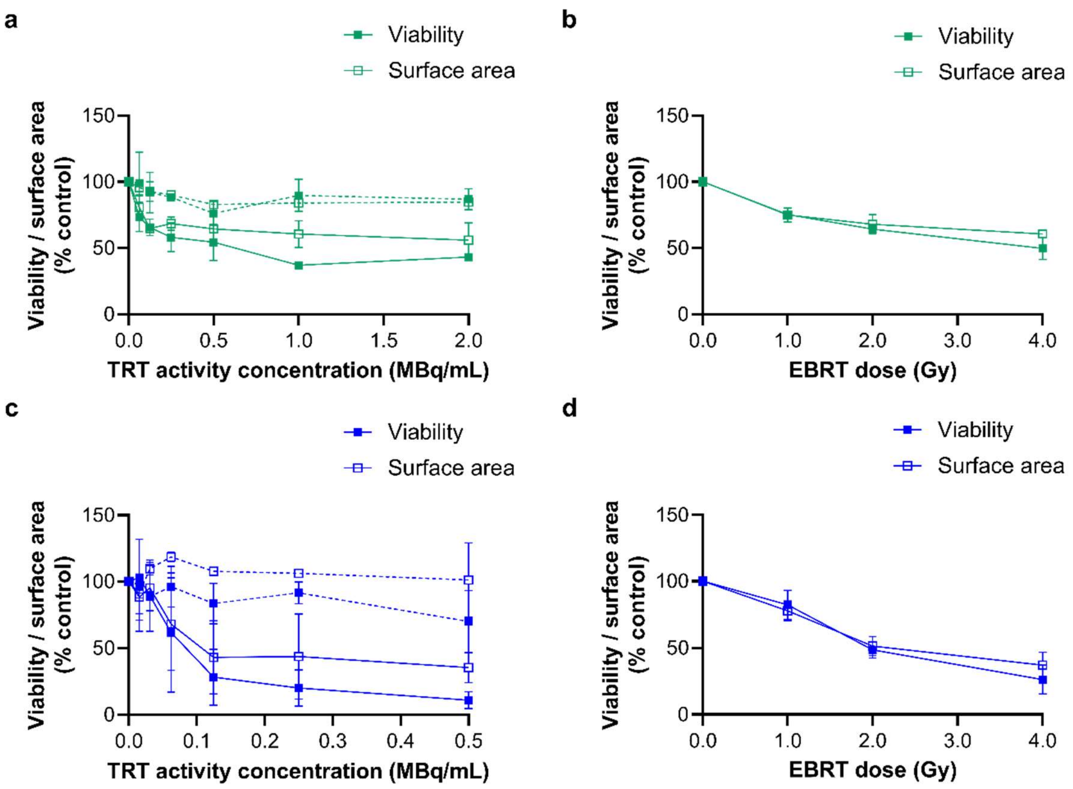

Supplemental Fig. 2 Viability and surface area of (a, b) LNCaP and (c, d) PC3-PIP bio-spheroids measured 6 days (a, c) after re-plating single cells incubated in suspension with several activity concentrations of [^{177}Lu]Lu-PSMA-I&T (solid lines) or [^{177}Lu]Lu-DTPA (dashed lines), the latter included as specificity control, or (b, d) after re-plating cells exposed to varying doses of EBRT in 2D setting following trypsinization. Data are normalized to untreated controls. The average surface area of untreated bio-spheroids was 0.43 ± 0.06 mm^2 and 0.37 ± 0.04 mm^2 for LNCaP and PC3-PIP, respectively. All data is the average of three independent biological experiments, except for PC3-PIP bio-spheroid cell viability (n = 4) and LNCaP/PC3-PIP bio-spheroid surface area (n = 2), both following [^{177}Lu]Lu-PSMA-I&T or [^{177}Lu]Lu-DTPA treatment. *2D = two-dimensional, TRT = targeted radionuclide therapy, EBRT = external beam radiation therapy, Gy = gray*

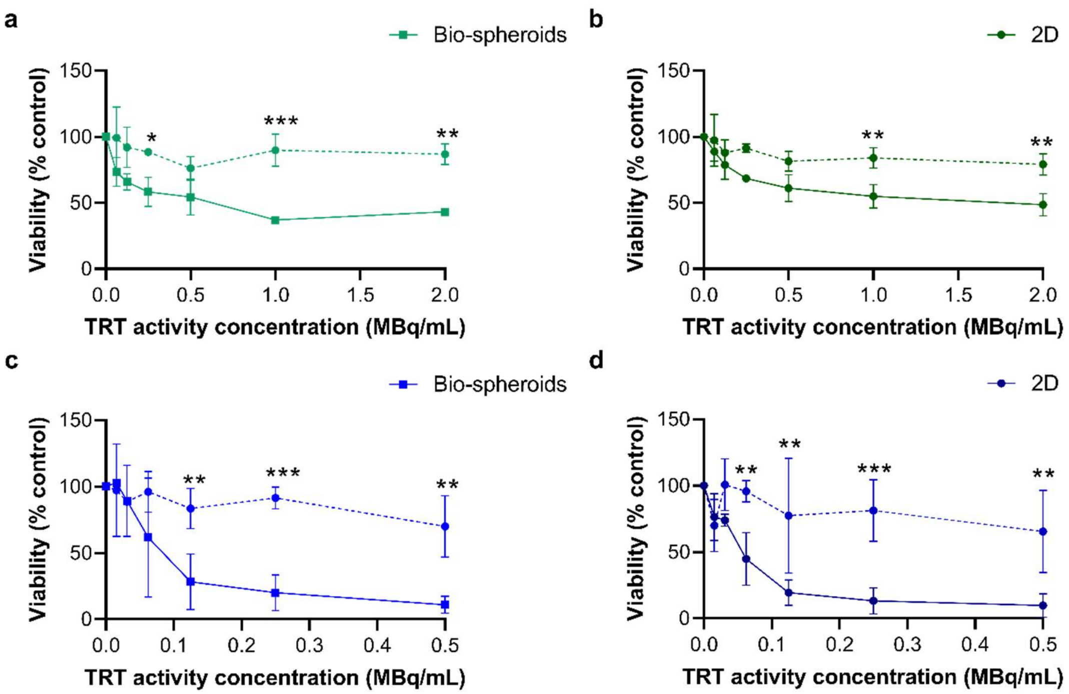

Supplemental Fig. 3 Viability of (a, b) LNCaP (n = 3) and (c, d) PC3-PIP (n = 4) cells cultured (a, c) as bio-spheroids or (b, d) in 2D setting measured 6 days after re-plating single cells incubated in suspension with several activity concentrations of [^{177}Lu]Lu-PSMA-I&T (solid lines) or [^{177}Lu]Lu-DTPA (dashed lines), the latter included as specificity control. Data are normalized to untreated controls. Asterisks indicate significant differences in cell viability after treatment with an identical activity concentration of [^{177}Lu]Lu-PSMA-I&T versus [^{177}Lu]Lu-DTPA, tested using a two-way ANOVA with Šidák correction. *2D = two-dimensional, TRT = targeted radionuclide therapy. * $p < 0.05$, ** $p < 0.01$, *** $p < 0.001$*

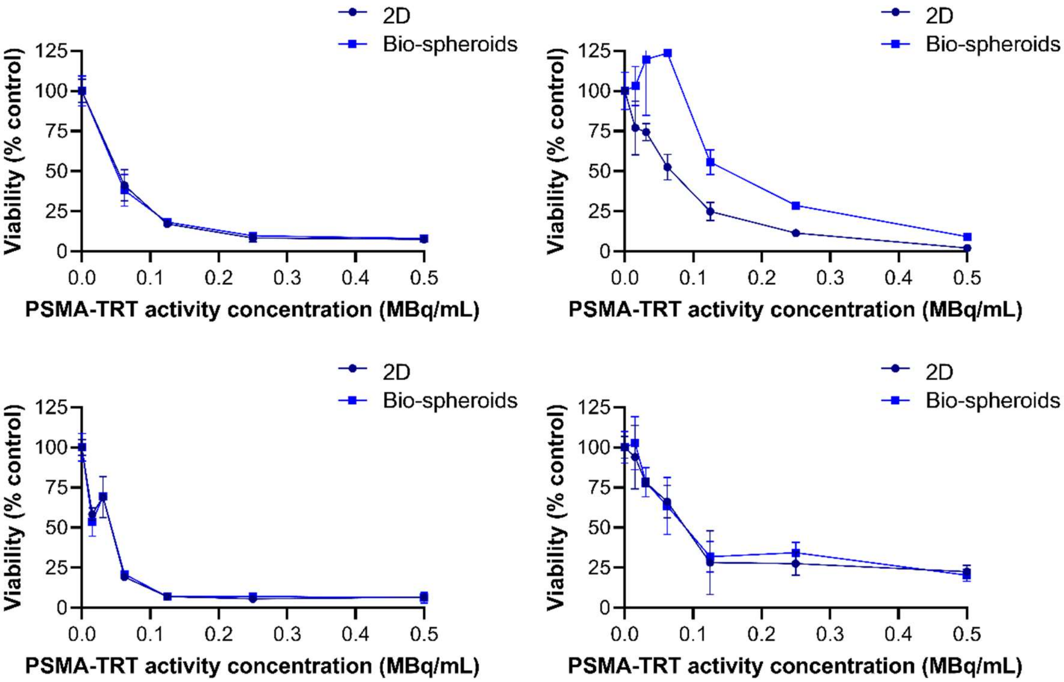

Supplemental Fig. 4 Viability of PC3-PIP cells cultured as bio-spheroids or in 2D setting measured 6 days after re-plating single cells incubated in suspension with several activity concentrations of [^{177}Lu]Lu-PSMA-I&T. Four independent experiments are provided and standard deviations represent technical variability. *2D = two-dimensional, PSMA-TRT = prostate-specific membrane antigen targeted radionuclide therapy*

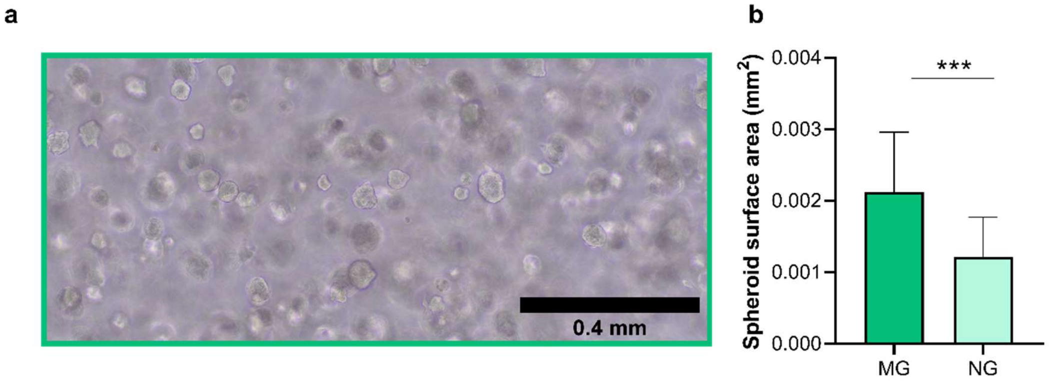

Supplemental Fig. 5 (a) Representative microscopic image of LNCaP MG-spheroids and (b) average LNCaP MG-spheroid (MG) and NG-spheroid (NG) surface area (n = 2, thirty spheroids per experiment), both obtained after 7 days of cell culture. Asterisk indicates significant difference in LNCaP MG- versus NG-spheroid surface area, tested using a non-parametric t-test. *MG = Matrigel, NG = Noviogel, *** $p < 0.001$*

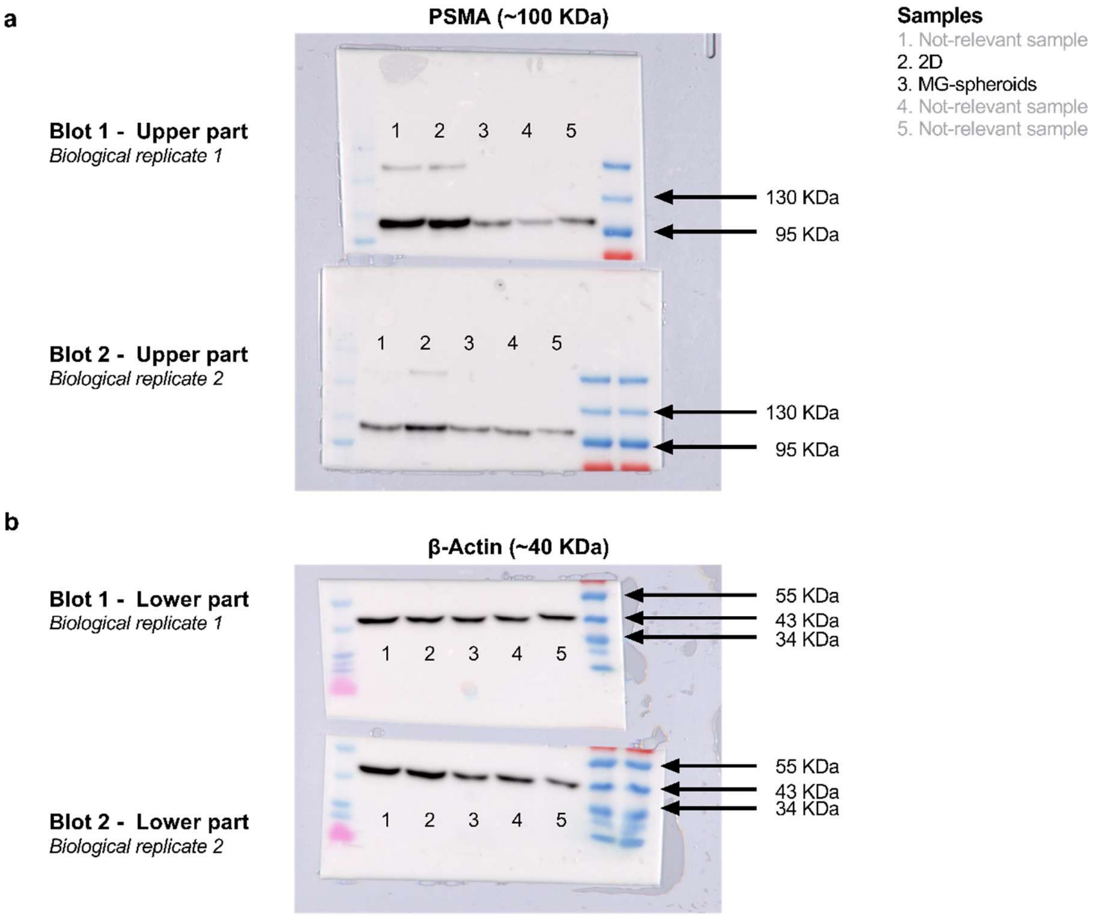

Supplemental Fig. 6 Unprocessed images of the western blot experiment measuring protein expression levels of (a) PSMA (~100 KDa) and (b) β-actin (~40 KDa) of LNCaP cells cultured in 2D setting or as MG-spheroids (n = 2). *2D = two-dimensional, MG = Matrigel*

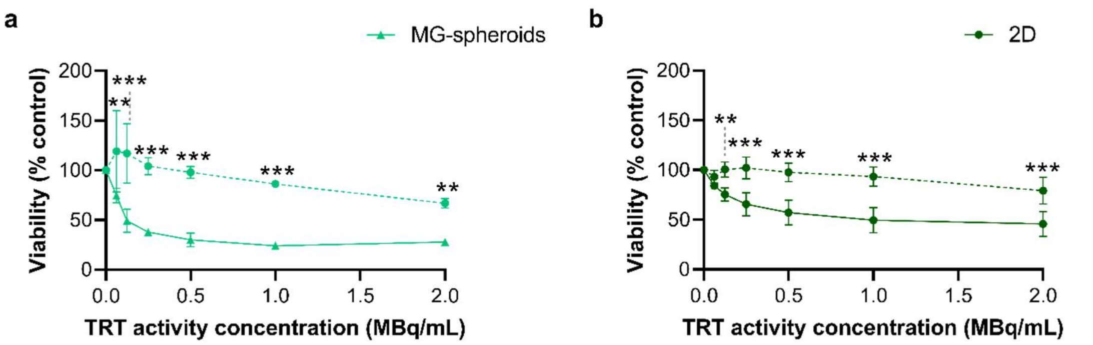

Supplemental Fig. 7 Viability of LNCaP cells cultured (a) as MG-spheroids (n = 3) or (b) in 2D setting (n = 4) measured 6 days after treatment with several activity concentrations of TRT using [^{177}Lu]Lu-PSMA-I&T (solid lines) or [^{177}Lu]Lu-DTPA (dashed lines), the latter included as specificity control. Data are normalized to untreated controls. Asterisks indicate significant differences in cell viability after treatment with an identical activity concentration of [^{177}Lu]Lu-PSMA-I&T versus [^{177}Lu]Lu-DTPA, tested using a two-way ANOVA with Šidák correction. *2D = two-dimensional, MG = Matrigel, TRT = targeted radionuclide therapy*

Supplemental Table 1 Primers used for RT-qPCR

Gene	Forward primer (5'-3')	Reverse primer (5'-3')
PSMA	GGAGAGGAAGTCTCAAAGTGCC	TGGTTCCACTGCTCCTCTGAGA
β-Actin	CTTCGCGGGCGACGAT	CCACATAGGAATCCTTCTGACC
GAPDH	CATCAAGAAGGTGGTGAAGC	ACCACCCTGTTGCTGTAG
HPRT1	CACTGGCAAAACAATGCAGACT	GTCTGGCTTATATCCAACACTTCGT

Supplemental Table 2 Antibodies used for western blot analysis

Protein	Primary antibody	Secondary antibody
PSMA	Rabbit anti-human monoclonal antibody ab133579 (1:50000, Abcam)	Goat anti-rabbit immunoglobulins/HRP P0448 (1:2000, DAKO)
β-Actin	Mouse anti-human monoclonal antibody MA1-140 (1:10000, Invitrogen)	Goat anti-mouse immunoglobulins/HRP P0447 (1:2000, DAKO)

Supplemental Table 3 Surface area measurements and/or viability data of LNCaP cells cultured in 2D or as bio-spheroids after [^{177}Lu]Lu-PSMA-I&T or [^{177}Lu]Lu-DTPA treatment, including mean values, standard deviations and *p*-values. Data is also shown in Fig. 2, Supplemental Fig. 2, and Supplemental Fig. 3.

Activity concentration (MBq/mL)	Culture method	Treatment	Surface area (% of control, mean ± SD)	Viability (% of control, mean ± SD)	*p* value *	*p* value **
**0.06**	2D<br>Bio-spheroids	[^{177}Lu]Lu-PSMA-I&T	N.A.<br>80.7 ± 8.6	88.7 ± 7.3<br>73.5 ± 11.0	0.2327	-
	2D<br>Bio-spheroids	[^{177}Lu]Lu-DTPA	N.A.<br>95.7 ± 5.5	97.2 ± 19.6<br>99.1 ± 23.3	-	0.8737<br>0.0616
**0.12**	2D<br>Bio-spheroids	[^{177}Lu]Lu-PSMA-I&T	N.A.<br>64.9 ± 0.1	78.6 ± 10.9<br>65.7 ± 6.3	0.4132	-
	2D<br>Bio-spheroids	[^{177}Lu]Lu-DTPA	N.A.<br>92.6 ± 7.4	87.8 ± 9.8<br>91.9 ± 15.2	-	0.8262<br>0.0527
**0.25**	2D<br>Bio-spheroids	[^{177}Lu]Lu-PSMA-I&T	N.A.<br>68.5 ± 5.1	68.5 ± 0.04<br>58.2 ± 11.0	0.7815	-
	2D<br>Bio-spheroids	[^{177}Lu]Lu-DTPA	N.A.<br>90.1 ± 2.7	91.3 ± 3.2<br>88.4 ± 0.1	-	0.0658<br>**0.0428**
**0.5**	2D<br>Bio-spheroids	[^{177}Lu]Lu-PSMA-I&T	N.A.<br>64.4 ± 2.1	61.0 ± 10.2<br>54.4 ± 13.8	0.9508	-
	2D<br>Bio-spheroids	[^{177}Lu]Lu-DTPA	N.A.<br>82.9 ± 1.9	81.5 ± 7.4<br>76.2 ± 8.9	-	0.0656<br>0.1571
**1.0**	2D<br>Bio-spheroids	[^{177}Lu]Lu-PSMA-I&T	N.A.<br>60.6 ± 10.1	54.9 ± 8.9<br>37.0 ± 0.1	0.1844	-
	2D<br>Bio-spheroids	[^{177}Lu]Lu-DTPA	N.A.<br>84.0 ± 6.4	84.0 ± 7.8<br>89.8 ± 12.3	-	**0.0035**<br>**0.0001**
**2.0**	2D<br>Bio-spheroids	[^{177}Lu]Lu-PSMA-I&T	N.A.<br>56.0 ± 13.3	48.5 ± 8.4<br>43.2 ± 0.4	0.9923	-
	2D<br>Bio-spheroids	[^{177}Lu]Lu-DTPA	N.A.<br>84.6 ± 1.7	79.1 ± 8.0<br>86.8 ± 7.9	-	**0.0020**<br>**0.0016**

N.A. = not available, SD = standard deviation, 2D = two-dimensional

** p-values indicate the statistical comparison of viability between 2D-cultured cells versus bio-spheroids following an identical activity concentration of [^{177}Lu]Lu-PSMA-I&T treatment.*

*** p-values indicate the statistical comparison of viability after treatment with an identical activity concentration of [^{177}Lu]Lu-PSMA-I&T versus [^{177}Lu]Lu-DTPA, both in 2D-cultured cells (upper) and bio-spheroids (lower).*

Supplemental Table 4 Surface area measurements and/or viability data of LNCaP cells cultured in 2D or as bio-spheroids after EBRT, including mean values, standard deviations and *p*-values. Data is also shown in Fig. 2 and Supplemental Fig. 2.

EBRT dose (Gy)	Culture method	Surface area (% of control, mean ± SD)	Viability (% of control, mean ± SD)	*p* value *
**1.0**	2D<br>Bio-spheroids	N.A.<br>74.9 ± 5.3	83.8 ± 1.9<br>75.3 ± 1.6	0.2095
**2.0**	2D<br>Bio-spheroids	N.A.<br>68.0 ± 7.4	63.9 ± 5.2<br>64.2 ± 3.6	> 0.9999
**4.0**	2D<br>Bio-spheroids	N.A.<br>60.6 ± 0.1	43.3 ± 9.4<br>49.8 ± 8.4	0.4365

N.A. = not available, SD = standard deviation, 2D = two-dimensional, Gy = gray, EBRT = external beam radiation therapy
** p-values indicate the statistical comparison of viability between 2D-cultured cells versus bio-spheroids following an identical dose of EBRT.*

Supplemental Table 5 Surface area measurements and/or viability data of PC3-PIP cells cultured in 2D or as bio-spheroids after [^{177}Lu]Lu-PSMA-I&T or [^{177}Lu]Lu-DTPA treatment, including mean values, standard deviations and *p*-values. Data is also shown in Fig. 3, Supplemental Fig. 2, and Supplemental Fig. 3.

Activity concentration (MBq/mL)	Culture method	Treatment	Surface area (% of control, mean ± SD)	Viability (% of control, mean ± SD)	*p* value *	*p* value **
**0.02**	2D<br>Bio-spheroids	[^{177}Lu]Lu-PSMA-I&T	N.A.<br>88.3 ± 17.3	76.5 ± 17.8<br>102.9 ± 0.3	0.5833	-
	2D<br>Bio-spheroids	[^{177}Lu]Lu-DTPA	N.A.<br>90.5 ± 14.8	70.0 ± 19.8<br>97.2 ± 34.8	-	0.9997<br>> 0.9999
**0.03**	2D<br>Bio-spheroids	[^{177}Lu]Lu-PSMA-I&T	N.A.<br>95.0 ± 16.9	73.9 ± 4.5<br>89.2 ± 26.9	0.9232	-
	2D<br>Bio-spheroids	[^{177}Lu]Lu-DTPA	N.A.<br>109.6 ± 4.6	100.7 ± 19.4<br>88.5 ± 0.5	-	0.5247<br>> 0.9999
**0.06**	2D<br>Bio-spheroids	[^{177}Lu]Lu-PSMA-I&T	N.A.<br>67.9 ± 34.7	44.8 ± 19.9<br>61.5 ± 44.8	0.7886	-
	2D<br>Bio-spheroids	[^{177}Lu]Lu-DTPA	N.A.<br>118.3 ± 3.3	95.8 ± 8.0<br>96.0 ± 15.3	-	**0.0048**<br>0.1476
**0.12**	2D<br>Bio-spheroids	[^{177}Lu]Lu-PSMA-I&T	N.A.<br>43.0 ± 27.5	19.3 ± 9.4<br>28.2 ± 20.9	0.9915	-
	2D<br>Bio-spheroids	[^{177}Lu]Lu-DTPA	N.A.<br>107.6 ± 0.6	77.5 ± 43.2<br>83.3 ± 15.1	-	**0.0010**<br>**0.0036**
**0.25**	2D<br>Bio-spheroids	[^{177}Lu]Lu-PSMA-I&T	N.A.<br>43.7 ± 32.0	13.3 ± 9.8<br>20.0 ± 13.6	0.9984	-
	2D<br>Bio-spheroids	[^{177}Lu]Lu-DTPA	N.A.<br>106.1 ± 2.9	81.3 ± 23.1<br>91.4 ± 8.2	-	**0.0001**<br>**0.0001**
**0.50**	2D<br>Bio-spheroids	[^{177}Lu]Lu-PSMA-I&T	N.A.<br>35.4 ± 11.4	9.7 ± 8.9<br>11.0 ± 6.3	> 0.9999	-
	2D<br>Bio-spheroids	[^{177}Lu]Lu-DTPA	N.A.<br>101.0 ± 28.0	65.5 ± 31.0<br>69.9 ± 23.1	-	**0.0017**<br>**0.0017**

N.A. = not available, SD = standard deviation, 2D = two-dimensional

** p-values indicate the statistical comparison of viability between 2D-cultured cells versus bio-spheroids following an identical activity concentration of [^{177}Lu]Lu-PSMA-I&T treatment.*

*** p-values indicate the statistical comparison of viability after treatment with an identical activity concentration of [^{177}Lu]Lu-PSMA-I&T versus [^{177}Lu]Lu-DTPA, both in 2D-cultured cells (upper) and bio-spheroids (lower).*

Supplemental Table 6 Surface area measurements and/or viability data of PC3-PIP cells cultured in 2D or as bio-spheroids after EBRT, including mean values, standard deviations and *p*-values. Data is also shown in Fig. 3 and Supplemental Fig. 2.

EBRT dose (Gy)	Culture method	Surface area (% of control, mean ± SD)	Viability (% of control, mean ± SD)	*p* value *
**1.0**	2D<br>Bio-spheroids	N.A.<br>77.8 ± 7.3	68.8 ± 12.3<br>82.3 ± 10.8	0.2035
**2.0**	2D<br>Bio-spheroids	N.A.<br>51.3 ± 7.2	31.6 ± 8.6<br>48.4 ± 6.3	0.0792
**4.0**	2D<br>Bio-spheroids	N.A.<br>37.1 ± 9.7	12.3 ± 3.6<br>26.1 ± 10.8	0.1854

N.A. = not available, SD = standard deviation, 2D = two-dimensional, Gy = gray, EBRT = external beam radiation therapy
** p-values indicate the statistical comparison of viability between 2D-cultured cells versus bio-spheroids following an identical dose of EBRT.*

Supplemental Table 7 Radiopharmaceutical uptake and *PSMA* mRNA expression levels of LNCaP cells cultured in 2D or as MG/NG-spheroids, including mean values, standard deviations and *p*-values. Data is also shown in Fig. 4.

**Culture method**	**Incubation time (h)**	**Uptake (% AD/ 100.000 cells)**	**Comparison**	***p* value**
**2D**	1	4.5 ± 2.1	1h vs 4h	0.0818
	4	9.0 ± 2.2	1h vs 22h	**< 0.0001**
	22	17.4 ± 4.2	4h vs 22h	**0.0021**
**MG-spheroids**	1	1.4 ± 0.6	1h vs 4h	0.4294
	4	4.0 ± 1.5	1h vs 22h	**0.0036**
	22	10.4 ± 4.6	4h vs 22h	**0.0248**
**NG-spheroids**	1	1.1 ± 0.2	1h vs 4h	0.0584
	4	5.1 ± 2.1	1h vs 22h	**0.0008**
	22	9.8 ± 2.2	4h vs 22h	**0.0185**
**2D**	22	17.4 ± 4.2	2D vs MG	0.0520
**MG-spheroids**		10.4 ± 4.6	2D vs NG	**0.0342**
**NG-spheroids**		9.8 ± 2.2	MG vs NG	0.9684
**Culture method**	***PSMA* mRNA expression (a.u.)**			***p* value**
**2D**	1.4 ± 0.004			**0.0003**
**MG-spheroids**	1.3 ± 0.013			

% AD / 100.000 cells = percentage added dose per 100.000 cells, 2D = two-dimensional, MG = Matrigel, NG = Noviogel, a.u. = arbitrary units, h = hours, PSMA = prostate-specific membrane antigen

Supplemental Table 8 Viability of LNCaP cells cultured in 2D or as MG-spheroids after [^{177}Lu]Lu-PSMA-I&T or [^{177}Lu]Lu-DTPA treatment, including mean values, standard deviations and *p*-values. Data is also shown in Fig. 5 and Supplemental Fig. 7.

**Activity concentration (MBq/mL)**	**Culture method**	**Treatment**	**Viability (% of control, mean ± SD)**	***p* value ***	***p* value ****
**0.06**	2D<br>MG-spheroids	[^{177}Lu]Lu-PSMA-I&T	84.1 ± 3.5<br>74.4 ± 7.3	0.7931	-
	2D<br>MG-spheroids	[^{177}Lu]Lu-DTPA	93.1 ± 6.5<br>119.1 ± 40.9	-	0.8272<br>**0.0065**
**0.12**	2D<br>MG-spheroids	[^{177}Lu]Lu-PSMA-I&T	75.4 ± 6.5<br>49.0 ± 11.6	**0.0081**	-
	2D<br>MG-spheroids	[^{177}Lu]Lu-DTPA	100.5 ± 7.8<br>116.9 ± 29.9	-	**0.0042**<br>**< 0.0001**
**0.25**	2D<br>MG-spheroids	[^{177}Lu]Lu-PSMA-I&T	65.7 ± 11.7<br>37.7 ± 0.1	**0.0046**	-
	2D<br>MG-spheroids	[^{177}Lu]Lu-DTPA	102.1 ± 11.0<br>104.0 ± 8.6	-	**< 0.0001**<br>**< 0.0001**
**0.50**	2D<br>MG-spheroids	[^{177}Lu]Lu-PSMA-I&T	57.1 ± 12.6<br>30.0 ± 6.9	**0.0016**	-
	2D<br>MG-spheroids	[^{177}Lu]Lu-DTPA	97.6 ± 9.5<br>97.9 ± 6.0	-	**< 0.0001**<br>**< 0.0001**
**1.0**	2D<br>MG-spheroids	[^{177}Lu]Lu-PSMA-I&T	49.4 ± 12.8<br>23.9 ± 3.7	**0.0031**	-
	2D<br>MG-spheroids	[^{177}Lu]Lu-DTPA	93.5 ± 9.6<br>86.0 ± 3.6	-	**< 0.0001**<br>**< 0.0001**
**2.0**	2D<br>MG-spheroids	[^{177}Lu]Lu-PSMA-I&T	45.8 ± 12.4<br>27.8 ± 3.2	0.0653	-
	2D<br>MG-spheroids	[^{177}Lu]Lu-DTPA	79.1 ± 13.5<br>66.7 ± 4.6	-	**< 0.0001**<br>**0.0035**

SD = standard deviation, MG = Matrigel, 2D = two-dimensional

** p-values indicate the statistical comparison of viability between 2D-cultured cells versus MG-spheroids following an identical activity concentration of [^{177}Lu]Lu-PSMA-I&T treatment.*

*** p-values indicate the statistical comparison of viability after treatment with an identical activity concentration of [^{177}Lu]Lu-PSMA-I&T versus [^{177}Lu]Lu-DTPA, both in 2D-cultured cells (upper) and MG-spheroids (lower).*

Supplemental Table 9 Viability of LNCaP cells cultured in 2D or as MG-spheroids after EBRT, including mean values, standard deviations and *p*-values. Data is also shown in Fig. 5.

**EBRT dose (Gy)**	**Culture method**	**Viability (% of control, mean ± SD)**	***p* value ***
**1.0**	2D<br>MG-spheroids	83.8 ± 1.9<br>82.6 ± 0.5	0.9985
**2.0**	2D<br>MG-spheroids	63.9 ± 5.2<br>51.1 ± 11.4	0.0648
**4.0**	2D<br>MG-spheroids	43.4 ± 9.4<br>40.0 ± 5.2	0.9426

SD = standard deviation, MG = Matrigel, 2D = two-dimensional, Gy = gray, EBRT = external beam radiation therapy

** p-values indicate the statistical comparison of viability between 2D-cultured cells versus MG-spheroids following an identical dose of EBRT.*
